# Mental health help-seeking by US college students with a diagnosis of psychosis

**DOI:** 10.1007/s00127-026-03102-7

**Published:** 2026-04-20

**Authors:** Clara Godoy-Henderson, Sara Shiv Denner, Michelle Flesaker, Janice Weinberg, Melissa Dalhoe, Sarah K Lipson

**Affiliations:** 1 School of Public Health, Boston University, Boston, MA USA; 2 Department of Psychiatry, University of Minnesota, Minneapolis, MN USA

**Keywords:** Psychosis, Help-seeking behaviors, Antipsychotic medication, Therapy, College student mental health

## Abstract

**Purpose:**

This study aims to examine the perceptions, beliefs, and attitudes that affect help-seeking behaviors among college students with a diagnosis of psychosis.

**Methods:**

Cross-sectional 2015–2024 national survey data from the Healthy Minds Study (HMS) were used to examine antipsychotic medication use, therapy/counseling utilization, and informal support engagement in the past 12 months among 2,819 U.S. college students with a diagnosis of psychosis. Descriptive statistics and adjusted logistic regression models were used to examine students’ help-seeking behaviors.

**Results:**

Approximately four-in-ten students with a diagnosis of psychosis reported antipsychotic medication use, eight-in-ten reported therapy/counseling utilization, and eight-in-ten reported engaging in informal supports. Eight-in-ten students agreed or strongly agreed that they needed help for their mental health in the past 12 months. As perceived need for help decreased, the use of antipsychotic medication, therapy/counseling, and engagement with informal supports also generally decreased. The belief that medication or therapy/counseling would not be helpful for mental health was generally associated with lower use of medication and therapy/counseling.

**Conclusions:**

Therapy and/or counseling in this population is highly utilized. A majority of students strongly agreed or agreed that they needed help for their mental health; however, six in ten students did not meet current recommended treatment guidelines for combined antipsychotic medication and therapy. High identified need for help and low service utilization, especially of antipsychotic medication, may indicate barriers to access.

## Introduction

Psychosis is a severe mental illness that affects a small percentage of the population, with global lifetime prevalence estimates of 7.49 per 1,000 persons [1]. Despite its overall low prevalence, psychosis is a major public health concern largely due to delayed initiation of mental health care. In the United States, the median duration of untreated psychosis to initiation of first-episode psychosis programs is 74 weeks (nearly 1.5 years) [2]. Prolonged untreated symptoms of psychosis can lead to greater symptom severity, a lower likelihood of remission, poor treatment response, and diminished social functioning [3–5]. Early intervention and continued service provision play a crucial role in improving health outcomes and quality of life for individuals with psychosis [6, 7]. With the onset of psychosis typically emerging in young adulthood, and with evidence showing that early intervention leads to better health outcomes [7–9], understanding help-seeking behaviors and mental health service use among young adults with psychosis is a key priority.

There is substantial evidence linking medication non-adherence to high relapse rates in people experiencing psychosis, a major clinical concern in this population [10, 11]. Medication non-adherence is linked to increased emergency room visits, prolonged hospital stays, higher risks of suicidal ideation, and greater risk of relapse [10–12]. While the existing literature highlights poor outcomes and high relapse rates associated with low treatment adherence in individuals diagnosed with psychosis, there is a research gap in understanding the factors, such as informal supports, that influence help-seeking behaviors and mental health service utilization [13, 14].

Early intervention services reduce the risk of treatment discontinuation and psychiatric hospitalization while improving outcomes related to overall quality of life, school or work involvement, symptom severity, employment and relapse rates [7, 8]. The efficacy of treatment when initiated early and adhered to for individuals with psychosis motivates a need to examine factors that encourage treatment initiation [15]. Examining mental health services utilization is especially important for the nearly 20 million college students in U.S. higher education [16]. This is particularly crucial as the traditional college years are characterized by young adults’ increasing independence in their health decision-making, including their use of health services. The traditional college years (young adulthood) are also an epidemiologically vulnerable time for the onset of many mental illnesses, including psychosis [9]. The average age of onset of psychosis (20.5 years) raises important questions about how U.S. college students with a diagnosis of psychosis access and navigate mental health services [9]. Many U.S. higher education institutions have rich resources to promote student well-being through organized social activities, counseling, peer support services, and or referrals [17, 18]. Exploring how college students diagnosed with psychosis engage with antipsychotic medication, therapy/counseling, and informal mental health supports can help identify patterns of service utilization and elucidate opportunities for potential earlier intervention to improve psychosis outcomes.

This study seeks to quantitatively examine the perceptions, beliefs, and attitudes that inhibit or facilitate the use of antipsychotic medication, therapy/counseling, and informal engagement (from friends, loved ones, roommates, campus staff, religious counselors, or support groups) among college students with a diagnosis of psychosis. Addressing the major knowledge gaps in mental health service utilization is a crucial first step toward reducing treatment delays that can improve outcomes for individuals with psychosis.

## Methods

### Data source and participants

This study utilizes the national Healthy Minds Study (HMS) cross-sectional data from 2015 to 2024 to examine mental health help-seeking among 2,819 college students with a diagnosis of psychosis. HMS is an annual web-based survey that collects demographic information, assesses mental health status, and measures help-seeking behaviors among U.S. college students. All postsecondary institutions in the United States are eligible to participate in the HMS. From each participating university, students are randomly invited to complete HMS. Students are over 18 years old and consent to complete the survey, which is collected via Qualtrics and is IRB-approved. The response rates among students by survey year were as follows: 23% (2015), 27% (2016), 23% (2017) 16% (2018), 16% (2019), 14% (Fall 2020), 13% (Winter 2020), 15% (2021), 12% (2022), 9% (2023), and 9% (2024). Previous research has published extensively on HMS data collection methods (Lipson et al., 2022).

Diagnosis of psychosis was determined by the HMS survey item, *“Have you ever been diagnosed with any of the following conditions by a health professional?”(Select all that apply)*. Students were included in the sample if they selected the response option “Psychosis”.

## Measures

### Primary outcomes

Therapy/counseling utilization in the past 12 months was determined by the survey item *“How many total visits or sessions for counseling or therapy have you had in the past 12 months?”*. If students indicated that they had at least one therapy or counseling session in the past 12 months, then they were classified as ‘yes’ for therapy/counseling use, and if they did not, they were classified as ‘no’. Antipsychotic medication use was measured by the survey item “*In the past 12 months*,* have you taken any of the following types of prescription medications?*” *(Select all that apply)*. If students selected “Antipsychotics,” then they were classified as ‘yes’ for antipsychotic medication use, and if they did not, they were classified as ‘no’.

Informal support engagement was determined by the survey question, “*In the past 12 months*,* have you received support for your mental or emotional health from any of the following sources?*” *(Select all that apply)*. Students were able to select “Roommate”, “Friend”, “Significant other”, “Family member”, “Religious counselor or other religious contact”, “Support group”, or “Other non-clinical sources, such as professor or staff member”. If any of those response options were selected, students were assigned a ‘yes’ to indicate informal support engagement, which was coded as a binary yes/no variable.

### Predictors

Reasons for seeking help were measured by the item, “*Earlier in this survey*,* you reported that you have taken medication and/or received counseling/therapy in the past 12 months for your mental or emotional health. Which of the following are important reasons why you received those services*?”. Students could select all that apply from the following reasons or factors that led them to seek care: “Self-initiated”, “Encouragement from family members”, “Pressure from family members”, “Encouragement from friends”, “Pressure from friends”, “Mandated or referred by a campus advisor”, “Someone other than a family member or friend, such as a health professional recommended or referred me to seek help”, and/or “Other”.

Perceived need for help was captured by survey item “*How much do you agree with the following statement? In the past 12 months*,* I needed help for emotional or mental health problems or challenges such as feeling sad*,* blue*,* anxious or nervous”.* Students were able to select “Strongly agree”, “Agree”, “Somewhat agree”, “Somewhat disagree”, “Disagree”, or “Strongly disagree”. Responses were coded numerically from 1 (“Strongly agree”) to 6 (“Strongly disagree”).

Perception of how helpful medication is was determined by the item “*How helpful on average do you think the medication would be for you if you were having mental or emotional health problems*?”. Students could select “Very helpful”, “Helpful”, “Somewhat helpful”, or “Not helpful”. Responses were coded numerically from 1 (“Very helpful”) to 4 (“Not helpful”).

Perception of how helpful therapy or counseling was measured by the item “*How helpful on average do you think therapy or counseling would be for you if you were having mental or emotional health problems?.* Students could select “Very helpful”, “Helpful”, “Somewhat helpful”, or “Not helpful”. Responses were coded numerically from 1 (“Very helpful”) to 4 (“Not helpful”).

### Statistical analysis

Using administrative data provided to the HMS team, survey non-response weights were applied to all analyses to account for the higher female response rate than males to more accurately represent each university’s female-male ratio. The weighted percent of participants’ age category, sex at birth, race/ethnicity, reasons for seeking help, perceived need for help, antipsychotic medication utilization in the past 12 months, therapy utilization in the past 12 months, perception of how helpful medication is, perception of how helpful therapy is, and survey year were examined by the total participant sample, students with antipsychotic medication use, students with therapy/counseling use, students with informal service use engagement, and students with no utilization in none of the three.

While some individuals may have used antipsychotic medication, therapy/counseling and informal supports, the primary focus of the analysis was to examine three sets of independent binary comparisons (1) students with a diagnosis of psychosis who report/do not report antipsychotic medication use, (2) students with a diagnosis of psychosis who report/do not report counseling/therapy use, and (3) students with a diagnosis of psychosis who report/do not report informal supports, rather than mutually exclusive service categories (e.g.,antipsychotic medication only therapy/counseling only, informal only). Logistic regression models with coefficients exponentiated to adjusted odds ratios (aOR) were used to assess the probability of antipsychotic medication, therapy/counseling use and informal service engagement in students with a diagnosis of psychosis. Logistic regression models were also used to estimate aORs of antipsychotic and therapy/counseling engagement with reasons for seeking help as the primary predictor, among help-seeking students Reasons for seeking help, antipsychotic medication, and therapy/counseling use in the past 12 month was assessed only among help seekers who engaged in antipsychotic medication and or therapy/counseling use due to survey skip logic. Predictors were chosen based on prior literature indicating a research gap on promotive factors improving medication and treatment adherence, low service utilization, and poor perception of treatment effectiveness in this population [19]. All models were adjusted for sex at birth (male and female), race (White, Asian, Hispanic/Latin(x), African American/ Black, or Other), and age categories (18–21, 22–25, 26–30, 31+). Analyses used a complete case approach, such that students with missing data on any variable included in the models were excluded from that analysis. All analyses were conducted using RStudio version 4.4.2.

## Results

### Characteristics of the sample (Table 1)

**Table 1:** Characteristics of the analytic sample of participants from the 2015–2024 waves of the Healthy Minds Study with self-reported psychosis diagnosis (*n* = 2819)

Unweighted sample size	All students with psychosis	Students with antipsychotic use	Students with therapy/counseling use	Students with Informal service use engagement	Students with no antipsychotic use, no therapy/counseling, and no informal support.
	*N* = 2819	*N* = 1,229	*N* = 2,175	*N* = 2,163	*N* = 93
	Weighted %	Weighted %	Weighted %	Weighted %	Weighted %
Age Category					
18–21	43.1	45.2	44.4	42.7	42.0
22–25	20.0	19.0	20.7	21.9	25.1
26–30	14.2	12.9	12.3	14.2	14.6
31+	21.8	22.9	22.7	21.2	18.3
Sex					
Male	52.2	52.6	54.3	54.8	51.4
Female	46.9	46.4	45.0	44.1	48.6
Intersex	0.9	1.0	0.7	1.0	0
Race					
White	57.8	57.8	58.6	58.9	61.3
Hispanic/Latin(x)	6.8	7.5	7.0	7.2	7.1
African American/ Black	10.4	9.9	10.7	9.7	5.4
Asian	4.5	4.6	4.3	4.8	3.8
Middle Eastern, Arab, or Arab American	1.9	0.4	0.4	0.5	0
Native Hawaiian / Alaskan / or Pacific Islander	4.6	0.7	0.7	0.7	0.9
Multiracial	16.4	16.5	16.2	15.7	18.9
Other	2.8	2.6	2.1	2.5	2.6
Survey year					
2015	1.6	1.0	1.3	1.6	0
2016	0.6	0.3	0.6	0.7	0.1
2017	3.7	3.3	3.5	3.9	4.5
2018	6.2	6.2	6.1	6.8	6.0
2019	6.9	7.2	6.5	6.9	10.5
2020	5.9	5.7	6.0	5.5	5.5
2021	12.4	11.6	11.6	12.1	16.1
2022	21.4	23.5	21.1	22.1	17.7
2023	21.5	21.7	21.8	22.0	27.9
2024	19.6	19.7	21.5	18.5	11.7
Perceived need for help					
Strongly agree	59.0	64.0	64.0	62.3	37.7
Agree	18.5	16.4	18.1	17.4	21.9
Somewhat agree	9.8	9.3	8.9	8.7	6.4
Somewhat disagree	2.4	1.5	1.5	2.0	5.1
Disagree	5.0	3.9	3.3	4.8	13.8
Strongly disagree	5.3	4.9	4.1	4.8	15.3
Antipsychotic Utilization in the past 12 months					
No Antipsychotic	55.7	0	48.4	53.9	100
Antipsychotic	44.3	100	51.6	46.1	0
Therapy Utilization in the past 12 months					
No Therapy	17.6	7.8	0	17.4	100
Therapy	82.4	92.2	100	82.6	0
Both Therapy and Antipsychotic Utilization in the past 12 months	39.6	89.5	51.6	42.0	0
Perception of how helpful medication is					
Very helpful	34.0	43.4	37.3	35.4	23.9
Helpful	28.2	31.4	29.8	27.3	33.2
Somewhat helpful	19.4	16.2	20.6	19.4	12.5
Not helpful	18.4	9.0	12.3	17.9	30.4
Perception of how helpful therapy is					
Very helpful	50.7	51.8	53.7	52.4	43.1
Helpful	26.6	26.9	27.8	26.3	24.6
Somewhat helpful	15.1	14.6	13.4	14.7	18.8
Not helpful	7.6	6.8	5.0	6.7	13.5

Our analytic sample included 2,819 students from 665 colleges and universities. Within this sample, just under half were between 18 and 21 years of age (43.1%), slightly over half were male (52.2%), and nearly six-in-ten were white (57.8%). Most students (62.2%) believed that medication would be very helpful (34.0%) or helpful (28.2%), compared to somewhat helpful (19.4.%) or not helpful (18.4%) for their mental health. Similarly, a majority of students with a diagnosis of psychosis strongly agreed (50.7%) or agreed (26.6%) that therapy or counseling would be very helpful for them if they were having mental or emotional health problems, while a minority (15.1%) disagreed or (7.6%) strongly disagreed. A complete description of student characteristics related to service utilization is reported in Table 1.

#### Characteristics related to service utilization

**Figs. 1:**
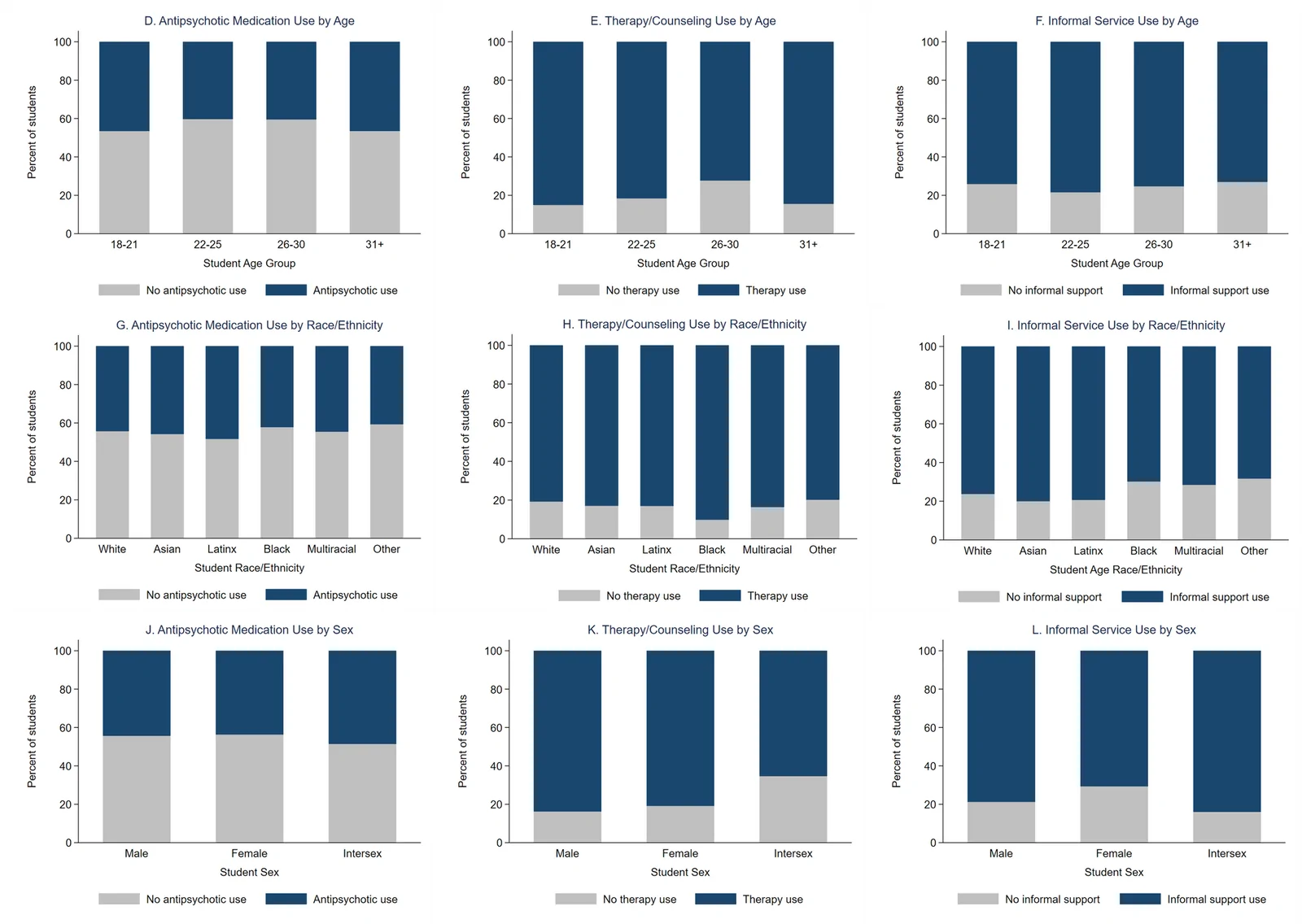
Percentage of students with a diagnosis of psychosis reporting antipsychotic medication use, therapy/counseling engagement, and informal support use by age, race/ethnicity, and sex

Of all students with psychosis, 44.3% reported taking antipsychotic medication in the past 12 months, 82.4% reported engaging in therapy/counseling in the past 12 months, and 74.7% reported engaging with informal supports. A total of 3.41% of students reported no engagement in antipsychotic medication, therapy/counseling, and informal supports. A total of 39.6% of students reported engaging in both antipsychotic medication and therapy or counseling in the past 12 months. Figure 1. presents stacked bar charts of the percent of students with reported antipsychotic medication use, therapy/counseling engagement, and informal service use by student age, race/ethnicity, and sex. Across most demographic groups, therapy/counseling and informal support engagement are higher than antipsychotic medication use.

**Figs. 2:**
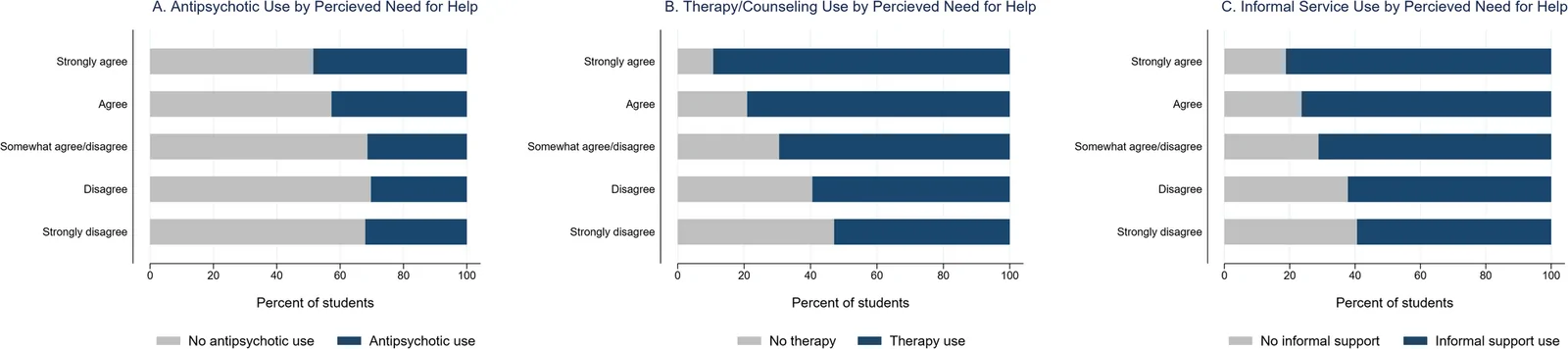
Percentage of students with a diagnosis of psychosis reporting antipsychotic medication, therapy/counseling, and informal support engagement by perceived need for mental health help

A majority (59%) of students with a diagnosis of psychosis strongly agreed that they needed help for emotional or mental health problems in the past 12 months. As seen in Fig. 2, as perceived need for help decreased, generally so did the use of antipsychotic medication, therapy/counseling, and informal support engagement.

#### Multivariable results for antipsychotic medication use, therapy/counseling utilization, and informal support engagement (Table 2)

**Table 2:** Adjusted logistic regression models assessing the associations between participant beliefs and use of antipsychotic medication, therapy/counseling, and informal services among participants with self-reported psychosis diagnoses in the 2015–2024 Healthy Minds Study

Perceived Need for Help^a, d, g^	Unweighted *n* in the total sample	OR Antipsychotic Use	95% CI	*p* value	OR Therapy/Counseling Use	95% CI	*p* value	OR Informal Service Use	95% CI	*p* value
	2,795									
Strongly Agree	1,730				ref			ref		
Agree	499	**0.68**	**0.47**,** 0.99**	**0.045**	**0.57**	**(0.35**,** 0.94)**	**0.027**	0.66	(0.41, 1.04)	0.073
Somewhat Agree	242	0.68	0.41, 1.12	0.136	**0.44**	**(0.25**,** 0.78)**	**0.005**	**0.59**	**(0.35**,** 0.99)**	**0.049**
Somewhat Disagree	77	**0.36**	**0.18**,** 0.74**	**0.005**	**0.19**	**(0.08**,** 0.41)**	**< 0.001**	**0.44**	**(0.22**,** 0.87)**	**0.018**
Disagree	119	0.57	0.29, 1.10	0.096	**0.21**	**(0.11**,** 0.40)**	**< 0.001**	0.83	(0.43, 1.60)	0.581
Strongly Disagree	128	0.70	0.37, 1.35	0.291	**0.31**	**(0.15**,** 0.63)**	**0.001**	0.59	(0.31, 1.11)	0.101
Perceptions of How Helpful Medication is^b, e, h^	1,345									
Very Helpful	488				ref			ref		
Helpful	368	0.73	0.47, 1.12	0.143	0.91	(0.49, 1.67)	0.747	0.74	(0.41, 1.32)	0.307
Somewhat Helpful	263	**0.41**	**0.25**,** 0.67**	**< 0.001**	0.83	(0.44, 1.5)	0.504	0.90	(0.50, 1.63)	0.733
Not Helpful	226	**0.19**	**0.11**,** 0.35**	**< 0.001**	**0.30**	**(0.15**,** 0.60)**	**0.001**	0.89	(0.47, 1.70)	0.725
Perception of How Helpful Therapy is^c, f, i^	1,345									
Very Helpful	657				ref			ref		
Helpful	372	0.94	0.61, 1.46	0.785	0.79	(0.41, 1.56)	0.503	0.75	(0.41, 1.36)	0.340
Somewhat Helpful	202	0.80	0.45, 1.42	0.475	**0.46**	**(0.23**,** 0.94)**	**0.034**	0.711	(0.39, 1.27	0.250
Not Helpful	113	0.73	0.38, 1.39	0.355	**0.21**	**(0.10**,** 0.44)**	**< 0.001**	0.51	(0.26, 1.01)	0.052

Note: Significant associations are in bold. All models adjusted for race (Asian, Latin(x)/Hispanic, African American/ Black, Other), Sex at birth (Male and Female), and age categories (18–21, 22–25, 26–30, 31+)

Footnotes:

^a^Model a assessed the association between the perceived need for help and antipsychotic use, adjusting for the same covariates (*n* = 2,756)

^b^Model b assessed the association between the perceived helpfulness of medication and antipsychotic use (*n* = 1,325)

^c^Model c assessed the association between the perceived helpfulness of therapy and antipsychotic use t (*n* = 1,324)

^d^Model d assessed the association between the perceived need for help and therapy/counseling use, adjusting for the same covariates (*n* = 2,594)

^e^Model e assessed the association between the perceived helpfulness of medication and therapy/counseling use, adjusting for the same covariates (*n* = 1,252)

^f^Model f assessed the association between the perceived helpfulness of therapy/counseling and therapy/counseling use, adjusting for the same covariates (*n* = 1,251)

^g^Model g assessed the association between the perceived need for help and informal support engagement (*n* = 2,756)

^h^Model h assessed the association between the perceived helpfulness of medication and informal support engagement (*n* = 1,325)

^i^Model i assessed the association between the perceived helpfulness of therapy and informal support engagement (*n* = 1,324)

Perceptions of need were correlated with antipsychotic use and therapy/counseling utilization among students with psychosis. Relative to students who strongly agreed that they needed help for their mental health, students who agreed that they needed help had 32% lower odds ofreporting antipsychotic use (aOR = 0.68, 95% CI 0.47–0.99) while students who somewhat agreed that they needed help had 64% lower odds of reporting antipsychotic use (aOR = 0.36, 95% CI 0.18–0.74). Compared to students who strongly agreed that they needed help for their mental health, students who agreed had 43% lower odds of reporting therapy/counseling use (aOR = 0.57, 95% CI 0.35–0.94), students who somewhat agreed had 56% lower odds (aOR = 0.44, 95% CI 0.25, 0.78), students who somewhat disagreed had 81% lower odds (aOR = 0.19, 95% CI 0.08, 0.41), students who disagreed had 79% lower odds (aOR = 0.21, 95% 0.11, 0.40), and students that strongly disagreed had 69% lower odds (aOR = 0.31 95% CI 0.15, 0.63).

Student perceptions of how helpful medication would be for them was significantly associated with antipsychotic medication and therapy/counseling. Compared to students who believed medication would be very helpful for their mental health, students who believed medication would be somewhat helpful had 59% lower odds of reporting antipsychotic medication use (aOR = 0.41, 95% CI 0.25–0.67), and students who identified medication has not helpful had 81% lower odds of antipsychotic medication use (aOR = 0.19, 95% 0.11, 0.35). Relative to students who identified therapy/counseling as very helpful, students who identified therapy as not helpful had 70% lower odds of reporting therapy/counseling use in the past 12 months (aOR = 0.30, 95% CI 0.15, 0.60).

Similarly, student perceptions of how helpful therapy would be for them was associated with engagement in therapy/counseling use in the past 12 monthsservice use. Relative to students who believed that therapy would be very helpful for their mental health, students who identified therapy as somewhat helpful had 54% lower odds of therapy/counseling use (aOR = 0.46, 95% CI 0.23, 0.94), and students who identified therapy as not helpful for their mental health had 79% lower odds of engaging in formal service (aOR = 0.21, 95% CI 0.10.–0.44).

Perceived need for help was associated with informal service use. Relative to students who strongly agreed that they needed help for emotional or mental health problems or challenges, students who somewhat agreed had 41% lower odds of engaging in informal supports (aOR = 0.59, 95% CI 0.35–0.99). Similarly, relative to students who strongly agreed to needing help for their mental health, students who somewhat disagreed had 57% lower odds of engaging in informal support (aOR = 0.44, 95% CI 0.22–0.87). Student perceptions of how helpful medication or therapy would be for their mental health was not associated with engagement in informal service use.

#### Multivariable results for types of supports associated with past-year antipsychotic medication and or therapy/counseling utilization among students engaged in formal service use (Table 3)

**Table 3:** Adjusted logistic regression models assessing the associations between reasons for seeking help and antipsychotic and or therapy/counseling use in the past 12 months among students engaged in formal services with self-reported psychosis diagnoses in the 2015–2024 Healthy Minds Study

Reasons for Seeking Help^j, k^	Unweighted *n* in the total sample	OR Antipsychotic Use	95% CI	*p* value	OR Therapy/Counseling	95% CI	*p* value
	1,734						
Decided to seek help on own	1,276	0.78	(0.52, 1.20)	0.248	1.81	(0.77, 4.27)	0.170
A friend encouraged me to seek help	319	0.95	(0.61, 1.50)	0.825	**4.89**	**(1.03**,** 23.1)**	**0.045**
A friend pressured me to seek help	126	1.00	(0.48, 2.08)	0.993	0.65	(0.16, 3.1)	0.634
A family member encouraged me to seek help	557	1.07	(0.72, 1.54)	0.713	1.23	(0.53, 2.86)	0.637
A family member pressured me to seek help	296	1.12	(0.70, 1.78)	0.629	0.84	(0.34, 2.03)	0.693
Someone other than a friend or family member encouraged me to seek help, such as a health professional	429	**1.49**	**(1.01**,** 2.20)**	**0.044**	1.26	(0.45, 3.51)	0.659
Campus advisor mandated or referred me to seek help	115	1.11	(0.61 2.01)	0.732	0.40	(1.07, 1.48)	0.659
I inquired more information about my options	25	0.62	(0.18, 2.11)	0.442	-	-	-
Other	104	0.80	(0.43, 1.51)	0.493	0.71	(0.17, 2.89)	0.632

Note: Significant associations are in bold. All models adjusted for race (Asian, Latin(x)/Hispanic, African American/ Black, Other), Sex at birth (Male and Female), and age categories (18–21, 22–25, 26–30, 31+). Reasons for seeking help series are binary variables; therefore, the reference group consists of those who did not select that item. The predictor inquiring more information about options was dropped from the model due to multicollinearity

Footnotes:

^j^Model j assessed the association between reasons for seeking help and antipsychotic use in the past 12 months (*n* = 1,724)

^k^Model k assessed the association between the reasons for seeking help and therapy/counseling in the past 12 months (*n* = 1,691)

Among help-seeking students engaged in antipsychotic medication and or counseling/therapy, encouragement from someone other than a friend or family member (such as a health professional) to seek help for mental health was also associated with antipsychotic use in the past 12 months. Relative to those who did not receive encouragement from someone other than a friend or family member, students who received encouragement to seek help (from someone other than a friend or family member, such as a health professional) had 1.49 times higher odds of taking antipsychotic medication in the past 12 months (95% CI 1.01–2.20). Encouragement from a friend to seek help for mental health was associated with therapy/counseling in the past 12 months. Relative to those who did not receive encouragement from friends to seek help, students who did had 4.89 times higher odds of therapy/counseling engagement in the past 12 months (95% CI 1.03–23.1); however, this finding should be interpreted with caution, given the large 95% CI.

## Discussion

This study examined the reasons why students with a diagnosis of psychosis seek help, their perceived need for help, and their perception of how effective medication and or therapy would be for their mental health, as predictors of formal service and informal support engagement using national HMS data from 2015 to 2024. This study of 2,819 students diagnosed with psychosis found that four-in-ten students report taking an antipsychotic medication in the past 12 months and eight-in-ten report utilizing counseling/therapy in the past 12 months, reflecting high rates of mental health service utilization. A total of four-in-ten students engaged in both antipsychotic medication and counseling/therapy in the past 12 months. This means—six-in-ten students are not meeting the recommended combination of both pharmacological and psychosocial treatment standard of care guidelines [20]. We found that negative perceptions of how helpful medication and or therapy/counseling would be for mental health was generally associated with lower odds of engagement in antipsychotic medication and therapy/counseling. Research suggests that negative perceptions, beliefs, and attitudes towards mental health treatment are likely modifiable [21]. Providing knowledge of symptoms, as well as information on the efficacy of medication or therapy/counseling, could be targeted through supportive relationships and psychoeducation, but its effects on treatment utilization needs to be further explored [21–23].

Studies have been able to capture antipsychotic prescribing trends in claims data [24, 25], but often fail to capture or take into account medication non-adherence rates which can be as high as 60% in this population [26], making the student self-reported medication and therapy or counseling in the past 12 months a unique comparator to prior research. Despite the high rates of medication non-adherence [26] and the strong engagement of therapy or counseling in the past 12 months, there remains an underutilized potential to leverage therapeutic interventions within this population. More than three-in-four students with psychosis strongly agreed or agreed that therapy or counseling would be very helpful for them if they were having mental or emotional health problems. This further underscores the importance of employing therapeutic interventions that are highly receptive in this population. Universities and clinicians alike can continue to capitalize on this population’s acceptability and strong engagement towards therapeutic interventions, which have been shown to reduce relapse, improve quality of life, and psychotic symptoms [27–29].

Overall, we found that those who are encouraged by friends or health professionals versus pressured or mandated as primary reasons for seeking help have higher odds of antipsychotic and or therapy/counseling use. This is an important implication given the institutionalization of mental illness that can result in mandated treatment [30]. Randomized controlled trials comparing mandated and voluntary patients with serious mental illness have found no significant differences in health service use outcomes, including risk of readmission and medication adherence [31]. Nonetheless, service engagement outcomes when treatment is self-initiated vs. mandated among individuals with psychosis remains largely unexplored [31]. Support systems, such as friends, play a crucial role in identifying early psychosis symptoms and help navigate mental health services, which may be an important factor in treatment initiation [32–34]. However, our finding of students who are encouraged by a friend to seek help, while statistically significant, had wide confidence intervals, and should be interpreted with caution. This warrants future research, particularly longitudinal studies to explore the long-term outcomes of therapy and antipsychotic engagement in individuals who are encouraged, pressured, or mandated to treatment.

We found that lack of perceived need for help was associated with lower odds of antipsychotic medication and therapy/counseling use in this sample. Notably, nearly 8 in 10 students reported that they needed help for their mental health in the past 12 months, but only 4 in 10 reported antipsychotic use in the past 12 months. Low utilization of antipsychotic medication, but high identified need for help, may indicate barriers to accessing antipsychotic medication. Previous research has identified barriers, including stigma, unpleasant medication side effects, communication gaps, and poor family support, as important contributors to poor medication utilization or adherence in patients with schizophrenia [35]. Thus, barriers to accessing antipsychotic medication may play an important role in utilization. This further underscores a potentially unmet need in this population to address and further examine barriers to accessing antipsychotic medication in future research.

The present study also examined the informal supports of students as an important resource affecting outcomes that is often not quantified or measured in studies. We found that the perceived need for help was also associated with informal engagement. Relative to students that strongly agreed that they needed help for emotional or mental health challenges, students who somewhat agreed or disagreed had significantly lower odds of engaging in informal supports. Support from friends, family, significant others, religious counselors, support groups, or campus may play a key role in supporting students’ overall well-being as well as treatment initiation. Informal supports, such as religious or spiritual healers, are often the first initial source of help in the initiation of symptoms, and are well accepted within this population [14, 34, 36]. In college and university settings, informal services and supports often come in the form of peer (i.e., student-to-student) or support groups. Peer support services promote positive vocational outcomes in first-episode psychosis programs, including higher odds of finding employment or enrolling in education [37].

Even though this is one of the largest known studies to our knowledge examining antipsychotic use, therapy/counseling use, and informal support engagement in students with psychosis, this study has limitations. While this study draws on national data from 2015 to 2024, this is a cross-sectional study and students were not followed over time. We therefore cannot establish causality. Similarly, given the cross-sectional study design, the temporality of various relationships cannot be established. For example, it is not possible to determine whether students utilize antipsychotic medication or therapy/counseling because they recognize a perceived need for mental health care or because they already hold positive views toward mental health treatment. With these survey data, it is not possible to determine the severity of their condition or whether students experienced multiple episodes of psychosis, a common attribute of the condition that may affect antipsychotic medication use, therapy/counseling utilization, and informal support engagement [10, 11]. Furthermore, we do not know the quality, intensity, or care delivery settings of the services students received. Further, some survey questions, such as perceptions related to perceived need and perceptions of how helpful medication and/or therapy/counseling would be if needed, captured students’ general attitudes toward identified need and mental health treatment overall. Therefore, perceptions regarding psychosis-specific treatments (e.g., attitudes toward antipsychotic medication, first-episode psychosis programs, or psychosis support groups) cannot be determined from these survey data. While students were randomly invited to complete the survey, students who completed the survey could have inherent characteristic differences between students who completed the survey and those who did not. For instance, because psychosis diagnoses were self-reported, there is an inability to know whether students with psychosis were more or less likely to respond to the survey. Secondly, while this study controlled for student characteristics including race, sex at birth, and age – residual confounding is a concern. For example, important characteristics such as the date of the last psychotic episode were not measured and are therefore missing from the analyses. Similarly, regression analyses used a complete case approach, such that students were included in the models only if they had no missing data on observed variables. This approach assumes that data are missing completely at random, and in this case, may underestimate estimates towards the null since we anticipate students who are less likely to complete the survey have greater adverse mental health concerns. Lastly, results are not adjusted for multiple testing, so we cannot rule out a statistically significant result occurring by chance.

## Conclusions

To our knowledge, this is one of the largest known studies examining antipsychotic medication use, therapy/counseling utilization, and informal support engagement in students with a diagnosis of psychosis. Therapy and/or counseling in this population is highly utilized and identified as being helpful for students’ mental health if they were having mental health challenges. As a result, therapeutic services on college campuses play an important role as an identified resource to college students with psychosis. Most students also identified that they needed help for emotional or mental health problems in the past 12 months. Encouragement from friends and or health professionals to seek treatment for mental health may promote help-seeking behaviors.

## Future research directions

Future research, particularly longitudinal studies, should try to distinguish the long-term mental health and service engagement outcomes when individuals are encouraged, pressured, or mandated to treatment. Research should also examine barriers to accessing antipsychotic medication in students and young adults, given the high self-identified need for mental health help, but low utilization of antipsychotic medication in this population.

## Data Availability

Data is publicly available online at [https://healthymindsnetwork.org/research/data-for-researchers/](https:/healthymindsnetwork.org/research/data-for-researchers).
